# Clinicopathological features for the prediction of immunosuppressive treatment responses in sarcoidosis-related kidney involvement: a single-center retrospective study

**DOI:** 10.55730/1300-0144.5907

**Published:** 2024-10-08

**Authors:** Ahmet Burak DİRİM, Vafa SÜLEYMANOVA, Özge HÜRDOĞAN, Özgür Akın OTO, Ayşe Serra ARTAN, Savaş ÖZTÜRK, Yasemin ÖZLÜK, Işın KILIÇASLAN, Halil YAZICI

**Affiliations:** 1 Division of Nephrology, Department of Internal Medicine, Faculty of Medicine, İstanbul University, İstanbul, Turkiye; 2 Department of Pathology, Faculty of Medicine, İstanbul University, İstanbul, Turkiye

**Keywords:** Sarcoidosis, granulomatous interstitial nephritis, glomerulonephritis, prognosis

## Abstract

**Background/aim:**

Sarcoidosis is a multisystem disorder that affects many organs, including the kidneys. This single-center retrospective study investigated the clinical, pathological, and laboratory findings of patients with kidney sarcoidosis who were treated with immunosuppressives.

**Materials and methods:**

Twenty-three patients with biopsy-confirmed kidney sarcoidosis were included. Demographic, clinical, pathological, and laboratory findings, in addition to the treatments and outcomes of 20 patients with at least one month of follow-up were evaluated.

**Results:**

The median age of the patients at the time of biopsy was 47 years (60.9% were female). The median baseline estimated glomerular filtration rate (eGFR) and proteinuria were 21.5 mL/min and 1 g/g or g/day, respectively. Nineteen of the 23 patients were diagnosed with nonglomerular disease (four had glomerular diseases). Extrarenal sarcoidosis was present in 86.7% of the patients. Granulomatous interstitial nephritis (56.5 %) and nephrosclerosis with intratubular calcific casts (17.4 %) were the two most common diagnoses. All the patients initially received 1 mg/kg/day steroids for kidney involvement. Although no statistical difference was observed in kidney function during the follow-up, steroids improved the eGFR in the first month compared with baseline in patients with nonglomerular diseases (p = 0.049). Eventually, 45% of the patients developed end-stage kidney disease, and 45% of cohort had a treatment response. Patients with higher baseline calcium levels (p = 0.03) and lower degrees of interstitial fibrosis/tubular atrophy (p = 0.043) had better kidney outcomes. Moreover, none of the patients with sarcoidosis-related secondary glomerular disease had a treatment response (p = 0.043).

**Conclusions:**

Hypercalcemia and lower interstitial fibrosis and tubular atrophy rates might be associated with better outcomes in sarcoidosis-related kidney involvement under immunosuppressive treatment. Moreover, late diagnosis, irregular follow-up, and glomerular disorders could be poor prognostic factors.

## 1. Introduction

Sarcoidosis is a systemic inflammatory disease that is usually associated with noncaseating granulomatous infiltration of the affected organs, mostly the lungs. The exact mechanism and pathogenesis have yet to be elucidated [1]. Lung involvement ranges from asymptomatic hilar lymphadenopathy to severe interstitial lung disease. In contrast, 8.7% of patients with sarcoidosis may present without lung involvement [2]. In addition, many extrapulmonary organs, including the kidneys, may be involved [3]. Isolated kidney involvement has also been reported in patients with sarcoidosis. [4]. Granulomatous tubulointerstitial nephritis (TIN) is the most common kidney pathological finding in patients with sarcoidosis. Moreover, nephrocalcinosis and kidney tubular injury due to hypercalcemia or secondary glomerulonephritis (GNs), including membranous glomerulonephritis, focal segmental glomerulosclerosis (FSGS), AA-type amyloidosis, and IgA nephropathy have been reported in the literature [5]. Some retrospective cohorts have included a limited number of patients with sarcoidosis-related kidney involvement [4–13]. Kidney involvement in sarcoidosis can cause end-stage kidney disease (ESKD), which may lead to mortality and morbidity, and corticosteroids are commonly used in kidney sarcoidosis. The most commonly used steroid treatment regimen is 1 mg/kg/day of prednisolone or methylprednisolone. Additionally, other immunosuppressives, such as azathioprine and mycophenolate could be used in corticosteroid refractory or dependent kidney sarcoidosis cases [5–7,11,12]. Nevertheless, predictors of kidney involvement in patients with sarcoidosis, and the prognosis of these patients, besides optimal treatment modalities, could have been partially illuminated due to the relative rarity of this situation. Therefore, it was aimed herein to investigate the clinical, pathological, and laboratory findings, in addition to predictors of treatment response, in patients with kidney sarcoidosis who were treated with immunosuppressives in this single-center retrospective study.

## 2. Materials and methods

### 2.1. Study population and patient evaluations

Twenty-three patients diagnosed with sarcoidosis, who underwent kidney biopsy between 2003 and 2023, were included in this retrospective study. Demographic, clinical, laboratory, and histopathological data were also recorded. Laboratory findings, including hemogram, serum creatinine, estimated glomerular filtration rate (eGFR) using the Chronic Kidney Disease Epidemiology Collaboration (CKD-EPI) equation [14], serum albumin, serum levels of corrected calcium with albumin, uric acid, phosphorus, C-reactive protein (CRP), angiotensin-converting enzyme (ACE) levels, spot urine protein-to-creatinine ratio (UPCR), and 24-h proteinuria at the time of kidney biopsy were evaluated. Histopathological findings, including pathological diagnosis, glomeruli count, global and segmental sclerotic glomeruli percentages, degree of interstitial fibrosis and tubular atrophy (IF/TA), interstitial inflammatory cell infiltration, intratubular calcific casts, and presence and degree of granuloma formation, were recorded. In addition, immunosuppressive treatments postbiopsy, mortality rates, and laboratory results at the last eGFR were evaluated in 20 patients with at least one month of follow-up data. In addition to the baseline data, the one month postbiopsy and last kidney function data were also noted. However, this retrospective study was based on patient files and the electronic healthcare system data of patients who had no regular follow-up. Therefore, some follow-up data, including clinical data, mortality, and the last eGFR, were extracted from electronic healthcare systems. The primary outcome was treatment response based on the last eGFR data. Complete recovery of kidney function was defined as an eGFR (CKD-EPI) >60 mL/min/1.73 m^2^ and proteinuria <0.5 g/g or g/day, and partial recovery of kidney function was defined as an eGFR >60 mg/dL and proteinuria 0.5–1 g/g or g/day, or an eGFR (CKD-EPI) >45 mL/min/1.73 m^2^ and proteinuria <0.5 g/g or g/day. Partial recovery was also defined as an increase in the eGFR by ≥25% of the baseline eGFR from the baseline to the last eGFR. Nonresponders were defined as individuals without complete or partial response. ESKD was defined as eGFR <15 mL/min/1.73 m^2^. The secondary outcomes were the development of ESKD and mortality.

### 2.2. Statistical analysis

In the descriptive statistics, median (interquartile range (IQR) 25%–75%) and frequency (percentage) values were used. The distribution of the variables was measured using the Kolmogorov–Smirnov test. The Mann–Whitney U test was used to analyze the quantitative independent data. The Wilcoxon and Friedman tests were used to analyze the dependent quantitative nonparametric data. The chi-squared test was used to analyze the qualitative independent data. The Fisher’s exact test was performed when the chi-squared test conditions were not met. IBM SPSS Statistics for Windows 27.0 (IBM Corp., Armonk, NY, USA) was used for the data analysis. p < 0.05 was considered statistically significant.

## 3. Results

### 3.1. Follow-up and demographic/clinical data of the patients

Fourteen of the 23 patients (60.9%) were female. The median age was 47 (min–max: 22–74) years. Moreover, 34.7% of the patients had a prior sarcoidosis diagnosis at the time of kidney biopsy. Twenty of the 23 patients had at least one month of follow-up data. Demographic, laboratory, histopathological, and clinical data of the study group are shown in Table 1. Twenty patients were divided into two groups, as the responders (complete or partial) and nonresponders. Nine of the 20 patients were responders (all of whom were partial responders).

### 3.2. Histopathological data of the patients

The patients were categorized as having glomerular and nonglomerular diseases, including granulomatous and nongranulomatous TIN and nephrosclerosis with tubular calcium deposits. Nineteen of the 23 patients were diagnosed with nonglomerular disease, and four patients had glomerular diseases. The most common diagnosis was granulomatous TIN (n = 13, 56.5%). Four patients (17.4%) had nephrosclerosis with intratubular calcific casts, without TIN. Two patients had FSGS (8.7%), and two patients (8.7%) were diagnosed with nongranulomatous TIN. AA-type amyloidosis and phospholipase A2 receptor (PLA2R)-positive membranous nephropathy (MN) were present in one patient (4.3%). Patients with glomerular diseases have lower treatment response rates than those with nonglomerular diseases (p = 0.043) (Tables 2 and S1). The degrees of tubulointerstitial inflammatory cell infiltration, granuloma formation, and IF/TA in biopsies, as well as the percentage of global and segmental sclerotic glomeruli, are shown in Table S1. No difference was observed in the pathological diagnosis between the responders and nonresponders. In contrast, none of the responders had moderate or severe IF/TA. The degree of IF/TA (not significant/mild vs. moderate/severe) showed statistical significance between the responders and nonresponders (p = 0.043). The percentage of global sclerotic glomeruli was higher in the nonresponders than in the responders. However, this difference was not statistically significant (p = 0.274). Other histopathological data for the responders and nonresponders are shown in Table 2.

### 3.3. Laboratory data of the patients

The baseline median creatinine level of the patients was 2.23 mg/dL at the time of biopsy. The baseline median eGFR and proteinuria levels of the patients were 26.5 mL/min/1.73 m^2^ and 1 g/g or g/day, respectively. Hypercalcemia (corrected serum calcium >10.4 mg/dL) was present in 17.4% of patients at the time of kidney biopsy. Eight patients had ACE levels at the time of biopsy, and for three of these patients, the ACE levels were elevated. Other laboratory data of the patient groups are shown in Table 1. The responders had higher corrected serum calcium levels than the nonresponders (p = 0.03). Correspondingly, the overt hypercalcemia rate was higher in the responders than in the other groups (p = 0.026). A comparison of the other laboratory data for the responders and nonresponders is provided in Table 2.

### 3.4. Extrarenal involvements

Of the patients, 69.6% had lung lymphadenopathy and 39.1% had extra-hilar lymphadenopathy (ultrasonography, CT, or histopathological examination). Two patients had histopathological nonnecrotizing granulomatous lymphadenitis; 17.4% cutaneous, 17.4% liver, 17.4% eye, 8.7% minor salivary gland, 4.3% arthritis, 4.3% bilateral parotitis, and 4.3% bone marrow involvement were present in the patient group during both the pre and postbiopsy follow-up. Isolated kidney sarcoidosis was present in three patients (13%). The diagnosis of isolated kidney sarcoidosis was based on granulomatous interstitial nephritis with noncaseating granulomas or nephrosclerosis with intratubular calcific casts and other findings such as fever and constitutional symptoms and/or increased ACE levels, and increased inflammatory markers and/or hypercalcemia, after excluding other granulomatous disorders. There was no statistically significant difference in extrarenal involvement between the patients with and without treatment response (Table 3).

### 3.5. Treatments

Twenty of the 23 patients had at least one month of postbiopsy follow-up. All 20 patients were treated with corticosteroids (initially 1 mg/kg/day of prednisolone with gradual tapering) postbiopsy. Moreover, azathioprine at 2 mg/kg/day (20%), hydroxychloroquine at 200 mg/day (10%), cyclosporine A (C0 through level aim: 50–150 ng/mL) (5 %), mycophenolic acid at 2000 mg/day (5%), and rituximab at 475 mg/m^2^/week for 4 weeks (5 %) were administered to patients with corticosteroid refractory sarcoidosis-related kidney involvement during follow-up (Tables 1 and 4).

### 3.6. Kidney recovery

None of the 20 patients with follow-up data had a complete treatment response. In addition, none of the patients with glomerular disorders exhibited a treatment response (p = 0.043 according to the chi-squared test for responsive vs. nonresponsive in glomerular vs. nonglomerular diseases). However, nine patients with nonglomerular diseases had a partial response (45%). In addition, nine of the 20 patients (45%) developed ESKD during the follow-up period. Changes in the eGFR during follow-up were not statistically significant, according to the Friedman test (p = 0.522). Wilcoxon signed rank test results for the baseline to one month postbiopsy eGFR data, one month postbiopsy to the last eGFR data, and baseline to the last eGFR data were also not statistically significant (p = 0.161, p = 0.588, and p = 0.940, respectively). However, the Wilcoxon signed rank test results for patients with nonglomerular diseases (16 patients) for baseline to one month postbiopsy were statistically significant (p = 0.049). The one month postbiopsy to month-to-last eGFR data and baseline-to-last eGFR data were not statistically significant (p = 0.453 and p = 0.140, respectively) (Figure). eGFR changes during follow-up were not statistically significant with the Friedman test in patients with nonglomerular disease (p = 0.185).

### 3.7. Mortality

Of the 20 patients, five (25%) died during the follow-up. Two patients developed ESKD due to kidney involvement in sarcoidosis. One of the non-ESKD patients died of high-grade B-cell lymphoma five years after the diagnosis of sarcoidosis (sarcoidosis-lymphoma syndrome). Two patients with partial kidney disease died during the study period (the etiology of mortality remains unknown).

Table 4 shows the clinical and pathological data as well as the outcomes of the 23 patients. Table S2 shows the summary of publications related to sarcoidosis with biopsy-proven native kidney involvement, which included more than 15 patients.

## 4. Discussion

The prognosis and the prognostic factors of patients with kidney sarcoidosis who were treated with immunosuppressives were investigated herein. Hypercalcemia and lower IF/TA rates were associated with better kidney outcomes after corticosteroid treatment, and glomerular diseases secondary to sarcoidosis had worse outcomes than nonglomerular sarcoidosis-associated kidney diseases. On the other hand, the absence of regular follow-up for some of the patients might lead to poor long-term patient and kidney outcomes.

Kidney involvement in sarcoidosis, a broad spectrum disease, is generally associated with multiorgan involvement. Increased soluble interleukin-2 receptor levels and decreased serum eGFR could be predictors of kidney involvement [12]. Granulomatous TIN was the most common kidney involvement in the current cohort, similar to previous publications [4,7–10,12]. In addition, nongranulomatous TIN, nephrosclerosis with intratubular calcific casts, and secondary glomerular diseases, including FSGS, AA-type amyloidosis, and PLA2R-positive MN, were present in the case series.

In the subgroup analysis, steroid treatment after diagnosis improved kidney function (eGFR) one month after steroid treatment in patients with nonglomerular disease. None of the patients with secondary glomerular diseases due to sarcoidosis responded to the treatment in the study (TIN and hypercalcemic nephrosclerosis were associated with better kidney function at the last follow-up than baseline data). This finding is consistent with that reported in the literature. Correspondingly, a study by Stehlé et al. [5], which included glomerular diseases due to sarcoidosis, had worse kidney and patient outcomes than the kidney sarcoidosis studies that included patients with TIN and hypercalcemic nephropathy [4,6–11] (Table S2). Glomerular diseases associated with sarcoidosis exhibit a broad spectrum of symptoms [5]. MN is the most common type of glomerular involvement in sarcoidosis. A recent study showed that PLA2R (38.9%) and neural epidermal growth factor-like-1 protein (NELL-1) (22.2%) positivity are common in sarcoidosis patients with MN. While the exact mechanism remains unknown, it was speculated that increased inflammation in sarcoidosis might cause increased glomerular expression of PLA2R and NELL-1 which might trigger antibody-mediated MN development [13]. Correspondingly, the patient herein with MN tested positive for PLA2R.

Histopathologically, none of the responders had moderate or severe IF/TA (p = 0.043). In addition, the responders had lower global sclerosis rates than the nonresponders, although this difference was not statistically significant (probably owing to the limited number of patients). In previous studies of kidney sarcoidosis, chronic lesions were associated with poor kidney outcomes [7,10]. In addition, most of the patients had multisystemic sarcoidosis, similar to the findings of previous studies (Table S2). The responders also had higher serum calcium levels at the time of the kidney biopsy. This finding was similar to the results reported by Rastelli et al. [10].

However, the patients herein had significantly poorer long-term kidney outcomes than those in the previous studies. This finding might be multifactorial. First, most of the patients had no history of sarcoidosis before the kidney biopsy (34.7%). Hence, the late diagnosis of multisystemic sarcoidosis may have been the reason for this. Accordingly, the nonresponders had more chronic histopathological lesions (IF/TA) than the responders. Second, most of the patients had irregular follow-ups, which may have resulted in inadequate treatment in the case of relapsing or persistent disease. Third, the study’s follow-up time was longer than that of some studies [8,11] regarding sarcoidosis-related kidney involvement, which could contribute to the declining eGFRs of the patients. Fourth, the kidney response criteria differed from those used in other studies. A more stringent response criterion was used than those in previous studies [4,7]. Fifth, the study group consisted of Turkish individuals, which might have caused different outcomes than in other studies.

Due to the retrospective nature of the study and the lack of regular follow-up, the extraction of data from patient files and electronic hospital systems were major limitations of this research. Relapsing disease was reported as a frequent problem in a study by Mahévas et al. [7]. Therefore, relapsing and refractory kidney diseases may have been inappropriately treated in the current cohort. In addition, the treatment compliance of the patients herein was indefinite. Detailed dosages of steroid treatments and side effects of immunosuppressive therapies could not be extracted due to limited data. Moreover, new-onset extra-kidney involvement might have been underdiagnosed because of irregular follow-up. However, this study, which included 23 patients with sarcoidosis-related kidney involvement, expands the spectrum of kidney sarcoidosis in the literature. Furthermore, it was also shown that hypercalcemia and tubulointerstitial involvement of sarcoidosis with early diagnosis (lower IF/TA rates) might be associated with better kidney outcomes under immunosuppressive treatments.

In conclusion, kidney sarcoidosis is a broad-spectrum disease generally associated with multisystem involvement. Moreover, hypercalcemia and lower IF/TA rates might be associated with better outcomes in kidney sarcoidosis, and the prognosis might be poor despite conventional immunosuppressive treatments in cases with late diagnosis, irregular follow-up, and secondary glomerular disorders. Therefore, multidisciplinary close follow-up of these patients should be an essential approach in clinical practice because it might require prolonged and long-term treatments.

## Supplementary

**Table S1:** Histopathological data of the patients.

Histopathological data	(n = 23)

Pathological diagnosis, n (%)	
Granulomatous TIN	13 (56.5)
Nongranulomatous TIN	2 (8.7)
Nephrosclerosis with intratubular calcific casts	4 (17.4)
FSGS	2 (8.7)
MN	1 (4.3)
AA-type amyloidosis	1 (4.3)

Tubulointerstitial inflammatory cell infiltration, n (%)	
No	5 (21.7)
Mild	9 (39.1)
Moderate	6 (26.1)
Severe	3 (13)

Granuloma formation, n (%)	
No granuloma	10 (43.5)
Mild/microgranuloma	3 (13)
Focal	7 (30.4)
Extensive	3 (13)

IF/TA, n (%)	
No	5 (21.7)
Mild	14 (60.9)
Moderate	3 (13)
Severe	1 (4.3)

Global sclerotic glomeruli (%), median (IQR 25–75)	12.5 (0–20)

Segmental sclerotic glomeruli (%), median (IQR 25–75)	0 (0–6.25)

**Table S2:** Summary of publications related to sarcoidosis with biopsy-proven native kidney involvement that included more than 15 patients.

	Number of patients	Kidney biopsy data	Baseline kidney function	Treatment	Follow-up time	Extra-kidney features	Kidney outcome	Mortality rates and other data
Rajakariar et al. (2006) [ 6]	39 patients, 20 patients had kidney biopsy	17 patients had granulomatous TIN (one of them had concurrent MN), 1 MCD 1 FSGS 1 IgAN	Mean creatinine of 17 patients with was 366 ± 299 μmol/L Baseline eGFR with MDRD 26.8 ± 14 mL/min	All 17 patients with TIN were treated with CS. 1 patient with concurrent MN was treated with CS + MYF 2 patients with relapses were treated with AZA and MYF	17 patients with TIN had a median of 84 months of follow-up time	17 patients with TIN had 47% of lung, 24% of lymph nodes, 35 % of uveitis, 24% of hypercalcemia, 18% of elevated ACE levels	First year eGFR of 17 patients with TIN was 49.6 ± 5.2 mL/min Last follow-up eGFR was 47.9 ± 6.8 ml/min	Steroid related side effects were observed in 3 of 17 patients (1 acute psychosis, 2 new-onset DM) 1 patient developed ESKD No mortality data was present
Mahévas et al. (2009) [ 7]	47 patients 19% of whom had prior known sarcoidosis diagnosis	37 patients had granulomatous TIN 10 patients had nongranulomatous TIN	Median eGFR was 20.5 mL/min	All patients were treated with CS. (median duration was 18 months) 10 of these patients received intravenous pulse MP 3 patients with kidney relapses were treated with AZA (n = 1) and MMF (n = 2)	Median Follow-up time: NA 41 of 47 patients had at least 1 year of follow-up	90% intrathoracic lesions 34% hypercalcemia 55% increased ACE levels 23.5% liver 21% uveitis 15% neuromuscular 10% skin 6% parotid 6% peripheral lymph nodes 6% cardiac 6% bone marrow 4% spleen 4% colon	60.5% complete response (Δ eGFR ≥50%) 23.5 % partial response (Δ eGFR = 50%–0%)	3 patients required hemodialysis at the end of follow-up. 17 patients had relapsing disease. At the end of follow-up, just 6 patients had steroid-free remission Initial degree of interstitial fibrosis had an inverse relationship with steroid response.
Stehlé et al. (2013) [ 5]	26 patients with sarcoidosis and glomerular diseases 11 patients had prior sarcoidosis	11 MN 6 IgAN 4 FSGS 3 MCD 2 proliferative lupus nephritis 6 patients had concurrent granulomatous TIN (2 MN, 2 IgAN, 1 FSGS, 1 MCD)	Mean baseline eGFR was 70.7 mL/min The mean baseline proteinuria was 5.7 g/day	25 of 26 patients received steroids for sarcoidosis. 18 of 26 patients received steroids and/or cytotoxic treatments were used to treat glomerular diseases 2 HCQ 2 AZA 1 MMF 2 CYC (patients with lupus nephritis)	Mean follow-up time was 101 months	9 biopsy confirmed lung 7 biopsy confirmed lymph node 3 biopsy confirmed skin 1 biopsy confirmed muscle 1 biopsy confirmed peritoneum involvements were present 22 of 26 patients had chest radiography confirmed disease 1 patient had uveitis. The mean number of affected organs was 2.6	9 complete remission (<0.3 g/day proteinuria) 7 Partial remission (>50% of reduction proteinuria and the last serum albumin > 3 g/L with 0.3–3 g/day proteinuria	6 patients (23%) had ESKD at the end of follow-up 3 patients (11.5%) expired
Bagnasco et al. (2014) [ 8]	51 patients had 56 native kidney biopsies	19 granulomatous TIN 8 nongranulomatous TIN 7 diabetic nephropathy 6 FSGS 6 chronic/advanced changes 3 immunocomplex nephritis 3 acute tubular injury 1 amyloid 1 MN 1 thin basement membrane disease 1 nonspecific	Limited follow-up data. 10 patients with granulomatous TIN had baseline mean creatinine 4.3 ± 2.6 mg/dL	Limited follow-up data. 10 patients had median 1.5 ± 0.8 years of treatment (Prednisone 50 ± 17 mg/day)	Limited follow-up data. 10 patients had median 3.0 ± 3.1 years follow-up	Limited follow-up data. 8 of 19 granulomatous TIN patients had hypercalcemia.	Limited follow-up data. Median last follow-up creatinine of 10 patients was 2.4 ± 1.6 mg/dL	1 of these 10 patients developed ESKD
Löffler et al. (2015) [ 4]	27 patients	12 nongranulomatous TIN 6 IgAN 5 granulomatous TIN 1 nephrocalcinosis 1 IgAN + granulomatous TIN Extensive tubular atrophy and fibrosis was present just in 1 patient	Baseline eGFR was 38 ± 21 ml/min	All patients received steroids (48% 1 mg/kg/day, 37% 0.5 mg/kg/day, 15% fixed 20 mg/day) Mean duration of treatment was 17.4 ± 10.9 weeks	The authors presented data over 2 years or more	15% of isolated kidney sarcoidosis 21 of 27 lung 4 of 27 cutaneous 4 of 27 extrathoracic LAP 3 of 27 hepatic 2 of 27 ophthalmologic 2 of 27 CNS 1 of 27 GI tract 1 of 27 peritoneal 5 of 27 hypercalcemia 10 of 21 elevated ACE 7 of 7 sIL2-R elevated 5 of 6 neopterin elevated	62.5% steroid response at the last follow-up. (Patients with impaired kidney function before steroids (CKD 2 or higher) response to treatment was defined as either complete when improvement of eGFR was 50% or more to baseline or partial when eGFR increased by 25%–49% to baseline level. Patients with any change in eGFR less than 25% or showing a decline in eGFR were considered non-responders. In patients with initially normal kidney functions response was defined as reduction of TPiU of 50% or more (complete response), respectively 25–49% to baseline (partial response). Any reduction in TPiU of less than 25% or an increment in protein secretion was considered non-responsive)	No kidney relapses were observed during follow-up. Mortality and ESKD/RRT data were not present
Kamata et al. (2018) [ 9]	16 patients, 63% whom had prior sarcoidosis diagnosis	13 granulomatous TIN 3 nongranulomatous TIN 2 kidney calcinosis (concomitant with TIN) 2 glomerular lesions (concomitant with TIN)	Mean eGFR 28.2 ± 16.1 mL/min/1.73 m ^2^	15 patients were treated with CS 1 patient was also treated with MTX	5.2 ± 4.6 years	100% lung 75% eye 31% heart 25% skin 13% neuromuscular 31% hypercalcemia 56% increased ACE	Last follow-up mean eGFR 43.7 ± 19.7 53% of patients had moderate to severe kidney dysfunction (Stages 3b and 4)	No patient had stage 5 kidney failure at the end of follow-up. No mortality was present in the cohort.
Rastelli et al. (2021) [ 10]	39 patients, 31 granulomatous TIN 5 nongranulomatous lesions with AKI 3 nephrotic syndrome	31 granulomatous TIN	Mean serum creatinine of patients with granulomatous TIN patients was 4.4 ± 2.3 mg/Dl Mean serum creatinine of patients with granulomatous TIN patients was 3.6 ± 2.3 mg/dL	36 patients were treated with CS. (9 patients: 0.5–1 mg/kg/day prednisone 5 patients: 0.75–0.8 mg/kg/day prednisone 20 patients: 1 mg/kg/day prednisone 1 patient: 8 mg/kg/day MP 1 patient: No data, 8 patients received IV pulse steroids before oral treatment)	22 patients with granulomatous TIN had more than 1 year follow-up. Median follow-up of these 22 patients was 77 months. Data on median follow-up of all study group was not available.	3 of 39 only kidney, Other patients had sarcoidosis with multiple organ/system involvement	Complete response (<10 ml/min eGFR difference between baseline (before AKI) and last eGFR) rate was Partial response (>10 mL/min eGFR difference between baseline (before AKI) and last eGFR with max GFR ≥25% of basal eGFR) was Non-response (the remaining of the patients) All 5 patients with nongranulomatous lesions with AKI had complete response 29 patients with granulomatous TIN had the last follow-up data. 13 of 29 (45 %) patients had complete response	No patient underwent HD treatment 3 patients expired Acute on set disease and hypercalcemia were associated with milder AKI and better kidney outcomes. Giant cells, severe infiltrates, and severe interstitial fibrosis were associated with worse kidney outcomes. Females had better endpoints than males.
Zhu et al. (2023) [ 11]	18 patients, 15 patients had kidney biopsy 5 patients had a history of extra kidney sarcoidosis (27.8 %)	10 granulomatous TIN 5 nongranulomatous TIN	Median eGFR was 30.36 mL/min/1.73 m ^2^	17 patients were treated with CS. (5 of them received pulse steroids-500 mg MP for 3 days) 2 patients were treated with tripterygium wilfordii glycosides in addition to CS 3 patients received MYF in addition to CS 1 patient was treated with CYC in addition to CS.	Median 24.07 months	1/18 (5.6%) isolated kidney sarcoidosis 15/18 (83.3%) lung 6/18 (33.3%) hypercalcemia 4/18 peripheral lymphadenopathy (22.2%) 2/18 skin (11.%) 1/18 eye (5.6%)	Last follow-up median eGFR 69.5 mL/min/1.73 m ^2^	No patient required HD at the end of follow-up 8 of 17 patients were maintained with CS No patient experienced relapses
Bergner et al. (2023) [ 12]	109 patients had probable or definite kidney sarcoidosis 90 patients had kidney biopsy	38 TIN 35 granulomatous TIN 24 nephrocalcinosis 11 secondary GN 1 nephrolithiasis	Median eGFR was 41.5 mL/min/1.73 m ^2^	109 kidney sarcoidosis cases were treated with 92% CS 38% AZA 7% MTX 5% MMF 1% HCQ 1% CYC 2% infliximab 1% adalimumab during follow-up	Long-term outcome of kidney sarcoidosis was not determined.	109 kidney sarcoidosis cases had 81% pulmonary 40% extrapulmonary LAP 25% liver 14% musculoskeletal 14% skin 13% cardiac 13% ophthalmic 9 % joint 7% neuro sarcoidosis 4% head, eye, ear, nose, throat involvement 39% increased ACE levels	Long-term outcome of kidney sarcoidosis was not determined.	sIL2-R levels were higher in kidney sarcoidosis patients than sarcoidosis patients without kidney involvement
Zubidat et al. (2023) [ 13]	18 patients with MN and sarcoidosis	18 MN biopsy specimens were analyzed with mass spectrometry 7 PLA2R (+) 4 NELL-1 (+) 1 THSD7A (+) 1 PCHD7 (+) 1 putative antigen Serpin B12 (+) No known target antigens were detected in 4 patients	Baseline median proteinuria was 9.8 g/24 hour, Median eGFR was 46.5 mL/min	No data	No data	No data	No data	No data

AKI: Acute kidney injury, CNS: central nervous system, CYC: cyclophosphamide, DM: diabetes mellitus, GI: gastrointestinal, MP: methylprednisolone, MTX: methotrexate, NELL-1: neural epidermal growth factor-like-1 protein, PLA2R: phospholipase A2 receptor 1, PCHD7: protocadherin-7, sIL2-R: soluble IL-2 receptor, THSD7A: thrombospondin type 1 domain-containing 7A.

## Figures and Tables

**Figure:**
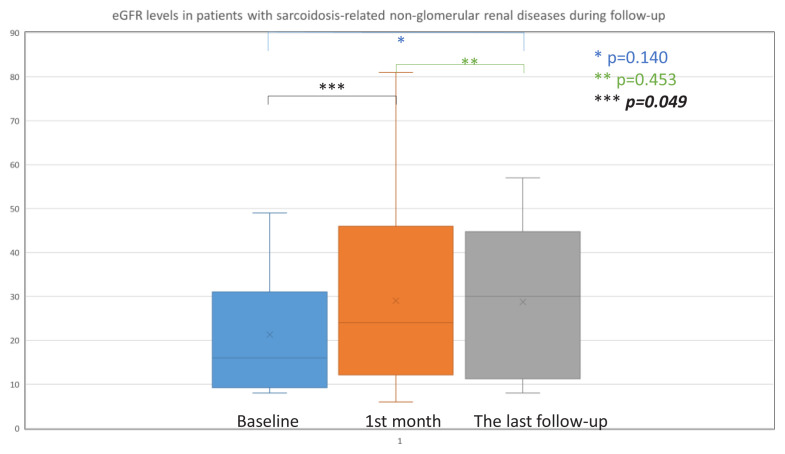
eGFR levels in patients with sarcoidosis-related nonglomerular renal diseases during follow-up.

**Table 1:** Demographic, clinical, follow-up, and laboratory data (at the time of biopsy) of the patients.

Demographic, laboratory, and clinical data	(n = 23)

Age (years), median (IQR 25–75)	47 (34–59)

Sex, female/male	14/9

Creatinine (mg/dL), median (IQR 25–75)	2.23 (1.88–4.38)

eGFR (CKD-EPI mL/min/1.73 m^2^), median (IQR 25–75)	26.5 (10.75–41.25)

Serum albumin (g/dL), median (IQR 25–75)	3.8 (3.35–4.15)

Calcium with corrected albumin (mg/dL) (median IQR 25–75)	9.8 (9.05–10.55)

Phosphorus (mg/dL) median (IQR 25–75)	3.8 (3.35–5.1)

Uric acid (mg/dL) median (IQR 25–75)	6 (5–6.3)

CRP (mg/L), median (IQR 25–75)	14 (3.43–42.5)

Hemoglobin (g/dL), median (IQR 25–75)	10.25 (8.4–12.98)

Proteinuria (g/g or g/day), median (IQR 25–75)	1 (0.25–2.56)

Prior history of sarcoidosis at time of biopsy, n (%)	8 (34.8)

Overt hypercalcemia (corrected Ca^++^ >10.4 mg/dL) at the time of biopsy, n (%)	4 (17.4)

Extrarenal features (including follow-up pre and postbiopsy), n (%)	Lung, including hilar LAP, 16 (69.6)
	Extra-hilar LAP, 9 (39.1)
	Liver, 4 (17.4)
	Skin, 4 (17.4)
	Eye, 4 (17.4)
	Minor salivary gland, 2 (8.7)
	Bone marrow granulomatous infiltration, 1 (4.3)
	Arthritis, 1 (4.3)
	Parotitis, 1 (4.3)

Long-term follow-up	n = 20

Follow-up time (months), median (IQR 25–75),	48 (17.25–72)

First month eGFR, median (IQR 25–75)	26.5 (13–49.75)

Last follow-up eGFR, median (IQR 25–75)	21.5 (11–39)

ESKD development, n (%)	9 (45 %)

Mortality rate, n (%)	3 (15 %)

Immunosuppressive treatments postbiopsy, n (%)	
Steroids	20 (100)
AZA	4 (20)
MMF	1 (5)
HCQ	2 (10)
RTX	1 (5)
CSA	1 (5)

AZA: Azathioprine, CSA: cyclosporine A, CRP: C-reactive protein, eGFR: estimated glomerular filtration rate, ESKD: end-stage kidney disease, FSGS: focal segmental glomerulosclerosis, HCQ: hydroxychloroquine, IF/TA: interstitial fibrosis and tubular atrophy, IQR: interquartile range, LAP: lymphadenopathy, MMF: mycophenolic acid, RTX: rituximab.

**Table 2:** Clinical, baseline laboratory, histopathologic and treatment data of the responders and nonresponders who received immunosuppressive treatments.

Parameters	Responders (n = 9)	Nonresponders (n = 11)	p-value

Age at the time of kidney biopsy, median (IQR 25–75)	51 (29.5–59)	46 (34–59)	0.909 ^m^

Sex, female/male	6/3	8/3	1.000 _F_

Creatinine, median (IQR 25–75)	3.1 (2.05–5.8)	1.9 (1.28–4.2)	0.138 ^m^

Uric acid, median (IQR 25–75)	5.9 (2.88–7.95)	6.05 (5.2–6.25)	0.948 ^m^

eGFR (CKD-EPI), median (IQR 25–75)	16 (9–33)	31 (13–45)	0.183 ^m^

Serum albumin, median (IQR 25–75)	3.9 (3.5–4.1)	3.7 (2.8–4.1)	0.596 ^m^

CRP, median (IQR 25–75)	17 (6.5–62)	14 (3.2–38.5)	0.560 ^m^

Hemoglobin, median (IQR 25–75)	10.2 (8.4–12.7)	10.2 (7.2–12)	0.954 ^m^

Proteinuria, median (IQR 25–75)	0.79 (0.29–1.08)	2.1 (1–7.5)	0.129 ^m^

**Calcium corrected with albumin, median (IQR 25–75)**	**10.8 (9.7–11.4)**	**9.35 (8.7–9.95)**	**0.03 ** **^m^**

Phosphorus, median (IQR 25–75)	3.8(3–4.3)	4.2 (3.63–5.65)	0.490 ^m^

**Hypercalcemia at the time of biopsy, n (%)**	**4 (44.4)**	**0 (0)**	**0.026 ** **_F_**

Follow-up time (months), median (IQR 25–75),	52 (25.5–91)	38 (7–72)	0.403 ^m^

Kidney limited sarcoidosis, n (%)	1 (11.1)	2 (18.2)	1.000 _F_

Prior history of sarcoidosis, n (%)	3 (33.3)	4 (36.4)	1.000 _F_

First month eGFR	36 (13–49.5)	24 (12.7–54)	0.939 ^m^

**Last eGFR**	**41 (31–51)**	**11 (9–14)**	**0.000 ** **^m^**

Mortality rate, n (%)	3 (33.3)	2 (18.2)	0.617 _F_

Treatments, n (%)			
Steroids	9 (100)	11 (100)	1.000 _F_
HCQ	1 (11.1)	1 (9.1)	1.000 _F_
AZA	1 (11.1)	3 (27.3)	0.591 _F_
MMF	0 (0)	1 (9.1)	1.000 _F_
RTX	0 (0)	1 (9.1)	1.000 _F_
CSA	0 (0)	1 (9.1)	1.000 _F_

Histopathological diagnosis, n (%)			
Granulomatous TIN	6 (66.7)	5 (45.4)	
Nongranulomatous TIN	1 (11.1)	1 (9.1)	
Nephrosclerosis	2 (22.2)	1 (9.1)	
FSGS	0 (0)	2 (18.2)	0.512 x**^2^**
MN	0 (0)	1 (9.1)	
AA-type amyloidosis	0 (0)	1 (9.1)	

**Glomerular disease subgroup**	**0 (0)**	**4 (36.4)**	**0.043 ** **_X_** **^2^**

Tubulointerstitial inflammatory cell infiltration, n (%)			
Not significant to mild	4 (44.4)	8 (72.7)	0.362 _F_
Moderate to severe	5 (55.6)	3 (27.3)	

Granuloma formation, n (%)			
No granuloma or mild/microgranuloma	5 (55.6)	7 (63.6)	1.000 _F_
Moderate to extensive	4 (44.4)	4 (36.4)	

**IF/TA, n (%)**			
No to mild	9 (100)	7 (63.6)	
Moderate to severe	0 (0)	4 (36.4)	**0.043 ** **_X_** **^2^**

Global sclerotic glomeruli (%), median (IQR 25–75)	0 (0–19.17)	16.6 (6.25–62)	0.274 ^m^

Segmental sclerotic glomeruli (%), median (IQR 25–75)	0 (0–18.75)	0 (0–12.5)	0.957 ^m^

F: Fisher’s exact test, m: Mann–Whitney U, MN: membranous nephropathy, TIN: tubulointerstitial nephritis, x^2^: chi-square (values in bold indicate statistically significant values).

**Table 3:** Comparison of the extrarenal organ involvement in the responders and nonresponders.

	Responders (n = 9)	Nonresponders (n = 11)	p-value
Lung, n (%)	5 (55.6)	3 (27.3)	0.642 _F_
Minor salivary gland, n (%)	1 (11.1)	1 (9.1)	1.000 _F_
Parotitis	1 (11.1)	0 (0)	0.450 _F_
Skin	2 (22.2)	1 (9.1)	0.566 _F_
Eye	2 (22.2)	1 (9.1)	0.566 _F_
Arthritis	1 (11.1)	0 (0)	0.450 _F_
Bone marrow	1 (11.1)	0 (0)	0.450 _F_
Liver	2 (22.2)	2 (18.2)	1.000 _F_
Extra-hilar lymphadenopathy	4 (44.4)	5 (45.5)	1.000 _F_

F: Fisher’s exact test.

**Table 4:** Biopsy, treatment, and follow-up data of each patient in the study group.

	Age at the time of biopsy	Sex	Serum Cr and eGFR at time of biopsy	Biopsy diagnosis	Immunosuppressant treatment postbiopsy during follow-up	Extrarenal organ involvements and other features of sarcoidosis (including the prebiopsy follow-up)	One month and last eGFR data	Follow-up time postbiopsy (months)	Mortality and other features
P-1	63	Female	4.2/10	Nongranulomatous TIN	CS	Lung, granulomatous hepatitis, extra-hilar lymphadenopathy	11 and 12 mL/min/1.73m^2^ (ESKD)	31	Tubulointerstitial inflammatory cell infiltration and severe IF/TA were present. Despite steroids, kidney functions were not ameliorated. The patient expired under HD treatment Nonresponsive disease
P-2	47	Female	1.9/31	Granulomatous TIN	CS	Not present (kidney-limited sarcoidosis diagnosis was established due to concurrent fever and constitutional symptoms after the exclusion of other granulomatous disorders)	18 and 11 mL/min/1.73m^2^ (ESKD)	58	Severe tubulointerstitial inflammatory cell infiltration with granulomas, and moderate IF/TA were present. Despite steroids, kidney functions deteriorated. HD patient. No mortality Nonresponsive disease
P-3	66	Female	1.2/45	FSGS	CS	Uveitis, and minor salivary gland involvement (biopsy was compatible with granulomatous sialadenitis)	35 and 9 mL/min/1.73m^2^ (ESKD)	72	At the time of biopsy microscopic hematuria and 3 g/day proteinuria. FSGS was attributed to sarcoidosis. After diagnosis, RAS blockade and corticosteroids were administered. Patient expired under HD treatment. Nonresponsive disease
P-4	34	Female	3.5/17	Granulomatous TIN	CS	Lung, extra-hilar lymphadenopathy	10 and 9 mL/min/1.73m^2^ (ESKD)	7	Tubulointerstitial inflammatory cell infiltration with extensive granulomas, and moderate IF/TA were present. Despite steroids, kidney functions deteriorated. HD patient. No mortality Nonresponsive disease
P-5	68	Male	3/20	Granulomatous TIN	No follow-up data	Lung	No follow-up data	NA	No follow-up data
P-6	23	Female	6.5/8	Granulomatous TIN	CS	Lung, extra-hilar lymphadenopathy	12.7 and 9 mL/min/1.73m^2^ (ESKD)	5	Severe tubulointerstitial inflammatory cell infiltration with granulomas. Despite steroids, kidney functions were not ameliorated. HD patient. No mortality Nonresponsive disease
P-7	61	Female	4/11	Granulomatous TIN	CS	Lung, extra-hilar lymphadenopathy	6 and 26 mL/min/1.73m^2^	70	Patient expired due to lymphoma (sarcoidosis lymphoma Syndrome developed 5 years after the sarcoidosis diagnosis) Partial response (responsive disease)
P-8	41	Male	NA	Nephrosclerosis with intratubular calcific casts	No follow-up data	Lung	No follow-up data	NA	No follow-up data
P-9	34	Male	2.1/40	Granulomatous TIN	CS, AZA	Lung, extra-hilar lymphadenopathy, and skin sarcoidosis	81 and 14 mL/min/1.73m^2^ ESKD	72	Tubulointerstitial inflammatory cell infiltration with granulomas Despite steroids and AZA, ESKD developed in 6 years HD patient. No mortality Nonresponsive disease
P-10	46	Female	1.9/31	Granulomatous TIN	CS, AZA, MMF	Not present (kidney-limited sarcoidosis diagnosis was established due to concurrent constitutional symptoms after the exclusion of other granulomatous disorders)	24 and 30 mL/min/1.73m^2^	68	No mortality Stable disease under immunosuppressives. Nonresponsive disease
P-11	59	Female	1.28/50	FSGS	CS, HCQ	Lung, extra-hilar lymphadenopathy, granulomatous hepatitis	54 and 17 mL/min/1.73m^2^	38	No mortality. At time of biopsy serum albumin:3,2 g/dL and UPCR:2,1. This patient had a history of Cushing syndrome and surkidneyectomy history one year before the sarcoidosis diagnosis. LGL (large granular lymphocyte syndrome) diagnosis was also present. FSGS was attributed to sarcoidosis. Nonresponsive disease
P-12	59	Female	1.8/30	AA type amyloidosis	CS	Lung	16.5 and 11 mL/min/1.73m^2^ (ESKD)	15	HD patient. No mortality At the time of biopsy serum albumin: 1.6 g/dL and 24-h proteinuria: 19 g/day. RAS blockade and steroids were started. This patient also had an asthma diagnosis. No other inflammatory disease history aside from sarcoidosis. Hence, AA-type amyloidosis was attributed to sarcoidosis. However, it progressed to ESKD. Nonresponsive disease
P-13	50	Female	1.97/29	Granulomatous TIN	CS	Lung, hypercalcemia	50 and 54 mL/min/1.73m^2^	44	No mortality. Last follow-up UPCR:0.1 Partial response (responsive disease)
P-14	32	Male	2.27/37	Granulomatous TIN	CS	Lung, skin (erythema nodosum)	49 and 57 mL/min/1.73m^2^	24	No mortality. Last follow-up UPCR:0.2. Partial response (responsive disease)
P-15	51	Male	1.6/49	Nephrosclerosis with intratubular calcific casts	CS	Hypercalcemia and increased ACE levels were present. No other organ involvement (kidney-limited)	51 and 48 mL/min/1.73m^2^	27	No mortality. Last follow-up UPCR:0.13 Partial response (responsive disease)
P-16	22	Male	1.93/48	Granulomatous TIN	CS	Lung, uveitis, skin, (pyogenic granuloma)	No follow-up data	No follow-up data	No follow-up data
P-17	45	Male	4.9/13	Nephrosclerosis with intratubular calcific casts	CS	Lung	29 and 8 mL/min/1.73m^2^ (ESKD)	2	No mortality. After ESKD development, patient underwent renal transplantation Nonresponsive disease
P-18	40	Male	0.8/112	Membranous GN (anti-PLA2R positive)	CS, AZA, CSA, RTX	Lung	110 and 14 mL/min/1.73m^2^ (ESKD)	91	No mortality. Despite multiple immunosuppressives, patient underwent PD due to resistant membranous GN. Nonresponsive disease
P-19	74	Male	5.8/9	Granulomatous TIN	CS, HCQ	Hypercalcemia and increased ACE levels. Minor salivary gland biopsy showed granulomatous sialadenitis. Bone marrow biopsy showed granulomatous infiltration	12 and 32 mL/min/1.73m^2^	108	Partial response (responsive) Mortality was present (Etiology?)
P-20	57	Female	2.2/24	Nephrosclerosis with intratubular calcific casts	CS	Hypercalcemia, arthritis, erythema nodosum (skin), uveitis	37 and 30 mL/min/1.73m^2^	74	Partial response (responsive) Mortality was present. (Etiology?)
P-21	25	Female	5.8/9	Granulomatous TIN	CS, AZA	Lung, extra-hilar lymphadenopathy, granulomatous hepatitis, bilateral parotitis,	24 and 33 mL/min/1.73m^2^	52	No mortality. Partial response (responsive)
P-22	55	Female	3.1/16	Non-granulomatous TIN	CS	Lung, extra-hilar lymphadenopathy	36 and 46 mL/min/1.73m^2^	204	No mortality. Last follow-up UPCR: 0.1 Partial response (responsive)
P-23	27	Female	6.6/8	Granulomatous TIN	CS	Extra-hilar lymphadenopathy, granulomatous hepatitis, uveitis, increased ACE	14 and 41 mL/min/1.73m^2^	2	No mortality. Patient also had splenomegaly and pancytopenia. Bone marrow biopsy showed no granuloma. Cytopenia was resolved after corticosteroid treatment. Partial response (responsive)

ACE: Angiotensin-converting enzyme, Cr: serum creatinine, CS: corticosteroids, GN: glomerulonephritis, HD: hemodialysis, IgAN: IgA nephropathy, LAP: lymphadenopathy, LGL: large granular lymphocytic leukemia, MCD: minimal change disease, MDRD: modification of diet in kidney disease, RAS: renin-angiotensin-aldosterone system, UPCR: spot urine protein to creatinine ratio.
